# Antimicrobial and Cytotoxicity Effects of Synthesized Silver Nanoparticles from *Punica granatum* Peel Extract

**DOI:** 10.1186/s11671-018-2731-y

**Published:** 2018-10-04

**Authors:** Sandhanasamy Devanesan, Mohamad S AlSalhi, Radhakrishnan Vishnu Balaji, Amirtham Jacob A Ranjitsingh, Anis Ahamed, Akram A Alfuraydi, Fulwah Y AlQahtani, Fadilah S Aleanizy, Ahmed H Othman

**Affiliations:** 1 Research Chair in Laser Diagnosis of Cancer, Department of Physics and Astronomy, College of Science, King Saud University, Riyadh, 11451 Kingdom of Saudi Arabia; 2 Department of Physics and Astronomy, College of Science, King Saud University, Riyadh, 11451 Kingdom of Saudi Arabia; 3 Stem Cell Unit, Department of Anatomy, College of Medicine King Saud, Riyadh, 11451 Kingdom of Saudi Arabia; 4 Deparment of Biotechnology Prathyusha Engineering College, Chennai, 602025 India; 5 Department of Botany and Microbiology, College of Science, King Saud University, Riyadh, 11451 Kingdom of Saudi Arabia; 6 Department of Pharmaceutics, College of Pharmacy, King Saud University, Riyadh, 11451 Kingdom of Saudi Arabia

**Keywords:** Silver nanoparticles, Pomegranate peel, Anticancer, Antibacterial, Phytosynthesis

## Abstract

To address the growing challenges from drug-resistant microbes and tumor incidence, approaches are being undertaken to phytosynthesize metal nanoparticles, particularly silver nanoparticles, to get remedial measure. In this study, an attempt has been made to utilize a major biowaste product, pomegranate fruit peel (*Punica granatum*), to synthesize silver nanoparticles. The silver nanoparticles (AgNPs) were synthesized using the aqueous extract of pomegranate peel. The formation of synthesized AgNPs was confirmed through UV-Vis spectroscopy, X-ray diffraction (XRD), transmission electron microscopy (TEM), scanning electron microscopy (SEM), and energy-dispersive X-ray spectroscopy (EDX) as well as through the change of the colorless aqueous solution to a dark brown solution. Using UV-Vis spectroscopy, the dark brown solution showed a Plasmon resonance band peak at 378 nm in UV-Vis spectroscopy after reacting for 24, 48, and 72 h. The XRD report revealed that the AgNPs had a cubic structure. The TEM and SEM report showed the nanoparticles were equally distributed in the solution, with a spherical shape and size ranging from 20 to 40 nm and with an average particle size of 26.95 nm. EDX imaging also confirmed the presence of AgNPs. The synthesized AgNPs were found to exhibit good antimicrobial effects on Gram-negative and Gram-positive bacteria, particularly the pathogens *Escherichia coli* (ATCC 25922), *Pseudomonas aeruginosa* (ATCC 27584), *Proteus vulgaris* (ATCC 8427), *Salmonella typhi* (ATCC 14028), *Staphylococcus aureus* (ATCC 29213), *Staphylococcus epidermidis* (MTCC 3615), and *Klebsiella pneumonia.* The cytotoxic effects of AgNPs were also tested against a colon cancer cell line (RKO: ATCC® CRL-2577™), and it was observed that the viabilities were 56% and 61% on days 3 and 5, respectively, with exposure to 12.5 μg of AgNPs. This simple, economic, and eco-friendly method suggests that the AgNPs biosynthesized using pomegranate peel extract may be a novel, potent solution for the development of a drug for colon cancer that also has antibacterial activity.

## Background

In the last few decades, there has been a growing amount of research on nanotechnology, particularly involving the green synthesis and characterization of nanoparticles, as nanoparticles less than 100 nm in size are ideal agents for drug delivery and biomedical applications [1]. The synthesis of nanoparticles plays an influential role in several fields, including nanotechnology, biotechnology, chemical processing, physical methodology, systems engineering, molecular motors, nanocrystals, and nanobiomaterials [2]. Three methods of nanoparticle production exist today—chemical, physical, and “green” routes, with the green route involving the employment of biological reducing agents, including plant extracts and microbial filtrates. The first two methods are often costly and generate toxic by-products, but the green nanosynthesis method has been recognized as an inexpensive and eco-friendly process [3–5].

In the green synthesis of NPs, plant constituents, including proteins, enzymes, and carbohydrates, are used to formulate nanoparticles that can easily interact with target biomolecules [6]. This approach to the synthesis of silver nanoparticles may play an important role in future treatments for various forms of cancer or other ailments that can be controlled by phyto-nanotechnology [7, 8]. Gram-negative bacteria, such as *Escherichia coli*, *Pseudomonas aeruginosa*, and *Proteus vulgaris*, and Gram-positive pathogens, such as *Staphylococcus aureus* and *S. epidermidis*, are responsible for most of the hospital-acquired infections [9]. Indeed, surgical infections, including pneumonia and bloodstream infections, are also due to the presence of Gram-positive and Gram-negative bacteria [10]. Plant-mediated synthesis of AgNPs can help in the development of effective antibacterial agents against microbial pathogens of public health relevance. Recently, it has been noted that synthesized AgNPs can have a synergistic relationship with the antibiotic levofloxacin, increasing the total antimicrobial activity [11]. Many researchers have reported that synthesized AgNPs contain well-known antimicrobial properties against Gram-positive and Gram-negative pathogens, as well as cytotoxic effects on different cancerous and normal cell lines [12–14]. In addition, AgNPs are highly efficient due to a high-surface-area-to-volume ratio, can easily disrupt, and have the ability to penetrate bacterial cells when compared to silver ions alone [13].

The current study is focused on the green synthesis of AgNPs using the aqueous extract of *Punica granatum* peel and on investigating their antimicrobial properties by using streak plates and minimum inhibition concentration (MIC) measurements after 24 h of incubation at 37 °C. The Gram-negative bacteria *E. coli* (ATCC 25922), *P. aeruginosa* (ATCC 27584), *P. vulgaris* (ATCC 8427), and *Salmonella typhi* (ATCC 14028) as well as the Gram-positive bacteria *Staphylococcus aureus* (ATCC 29213), *S. epidermidis* (MTCC 3615), and *K. pneumoniae* were studied to test the potential growth inhibition by synthesized AgNPs. Furthermore, the cytotoxic effects on a colon cancer cell line (RKO: ATCC® CRL-2577™) were tested and showed a cell viability rate of 56% on day 3 and 61% on day 5 with a dose of 12.5 μg of AgNPs.

## Methods

### Preparation of the Peel Extract

One kilogram of Saudi pomegranate fruits (*Punica granatum*—cultivated in the Taif region of the Kingdom of Saudi Arabia) was purchased from the supermarket in Riyadh, Saudi Arabia. The fruits were washed several times with tap water and then with double-distilled water (DDH_2_O). After washing, the peel was carefully removed. The pomegranate peel was rinsed thoroughly with DDH_2_O to avoid any surface contamination and allowed to dry completely at room temperature. Finally, the peel was ground into a fine power. Ten grams of the fine powder was soaked in 100 mL of DDH_2_O for 24 h at room temperature. The resulting mixture was filtered using Whatman No. 1 filter paper to acquire the aqueous extract. The entire process was performed in sterilized conditions.

### Synthesis Process of AgNPs

Silver nitrate (AgNO_3_; 0.1 mM) was mixed with 250 mL of DDH2O. Then, ten milliliters of aqueous pomegranate peel extract was added, and the solution was thoroughly mixed using a shaking incubator for 5 min. The reaction mixture was found to change its color from a colorless solution to a brown-colored solution after 24 h, indicating the reduction of the silver ions into silver nanoparticles. The nanoparticle solution was then centrifuged at 15,000 RPM for 15 min, and the process was repeated four times. Finally, purified AgNPs were collected, and further assays were performed to analyze the characteristics and biological activities of the synthesized NPs. The excess peel extract was stored at 4 °C for further analysis.

### Characterization of the AgNPs

The reduction of silver ions by the pomegranate peel aqueous extract was monitored using a Perkin Elmer Lambda 950 UV/Vis/NIR spectrophotometer 24, 48, and 72 h after the start of the reaction from 200 to 800 nm and at a resolution of 1 nm. XRD patterns were obtained by a PANalytical X-ray diffractometer capable of scan speeds ranging from 20 to 50 with 2*θ* and were used to determine the crystalline structure of the silver nanoparticles.

Surface topographical and composition analyses of the AgNPs were accomplished using TEM analysis performed on a JEOL JEM-1230 (JEOL, Tokyo, Japan) and JSM 6380 LA SEM, with a resolution of 3.0 nm. The elemental analysis of the AgNPs was performed by energy-dispersive X-ray spectroscopy (EDX) using a JED 2200 series (Jeol).

### Antibacterial Studies

#### Bacterial Suspension Preparation

Bacterial strains *E. coli* (ATCC 25922), *P. aeruginosa* (ATCC 27584), *P. vulgaris* (ATCC 8427), *S. typhi* (ATCC 14028), *S. aureus* (ATCC 29213), *S. epidermidis* (MTCC 3615), and *K. pneumoniae* were obtained from King Khalid Hospital, Riyadh, Kingdom of Saudi Arabia. A rapid identification of bacterial cells was performed according to previously published methods [15]. All identified cultures were transferred onto agar media and stored at − 20 °C until needed for the study. At that point, each bacterial strain was inoculated into sterile nutrient agar and incubated at 37 °C for 24 h. The suspension (10^6^ CFU/mL) was prepared by transferring a loop of inoculum from the 24-h incubated culture into 5 mL of nutrient broth and incubating it at 37 °C for 2 h.

#### Antimicrobial Assays

Antimicrobial activity assays were carried out using an agar well diffusion method [16]. A sterile swab was moistened with fresh bacterial suspension and spread on a solid, sterile Muller-Hinton agar plate. Wells were made in the agar plate using a cork borer. Different concentrations (25, 50, 75, and 100 μL) of synthesized nanoparticle suspension were poured into each consecutive well. All plates were incubated at 37 °C for 24 h. A zone of inhibition was measured (mm) around each well in every incubated plate. For each experiment, three replicates were performed [17].

#### Cell Proliferation Analysis

The effect of AgNPs on cellular proliferation was evaluated using an Alamar Blue assay as described previously [12].

In brief, 0.005 × 10^6^ cells/well were seeded in 96-well plates with different concentrations (100–0.3 μg/mL) of AgNPs and incubated for 2 to 5 days at 37 °C. The medium, DMEM, was supplemented with 4500 mg/L d-glucose, 4 mM l-glutamine, 110 mg/L sodium pyruvate, 10% fetal bovine serum (FBS), 1× penicillin-streptomycin, and non-essential amino acids (all purchased from Gibco-Invitrogen, USA). Control wells were treated with media alone, and cell proliferation was measured on day 3 and day 5. At these time points, Alamar Blue (1:10) was added to each well, and the plates were incubated at 37 °C for 4 h; then, the plates were read using a spectrophotometric microplate reader (Biotek Synergy 2; Biotek Instruments, USA), and the relative fluorescence unit (RFU) was recorded.

#### Cell Apoptosis/Necrosis Analysis

To determine apoptosis/necrosis, the cells were treated with AgNPs at different concentrations (25–1.5 μg/mL). On day 5, the cells were stained with a dual fluorescent staining solution (1 μL) containing 100 μg/mL AO (acridine orange) and 100 μg/mL EtBr (ethidium bromide) (AO/EtBr, Sigma, St. Louis, MO). The stained cells were exposed to an AO/EtBr (1:100) dye solution for 1 min and observed using a Nikon Eclipse Ti fluorescence microscope. The results were compared with the experimental control. AO/EtBr, a combination of two dyes, helps to visualize cells with aberrant chromatin organization. The differential uptake of AO/EtBr allows the identification of viable and non-viable cells. Particularly, the AO was used to visualize the number of cells that had undergone apoptosis.

#### Statistical Analysis

Statistical analyses and graphing were performed using Microsoft Excel 2010 and GraphPad Prism 6.0 software (GraphPad, San Diego, CA, USA). *P* values were calculated using one-way ANOVA multiple comparisons. Antimicrobial data analysis for different concentrations was tested with a significance level of *P* < 0.05.

## Results and Discussion

**Fig. 1:**
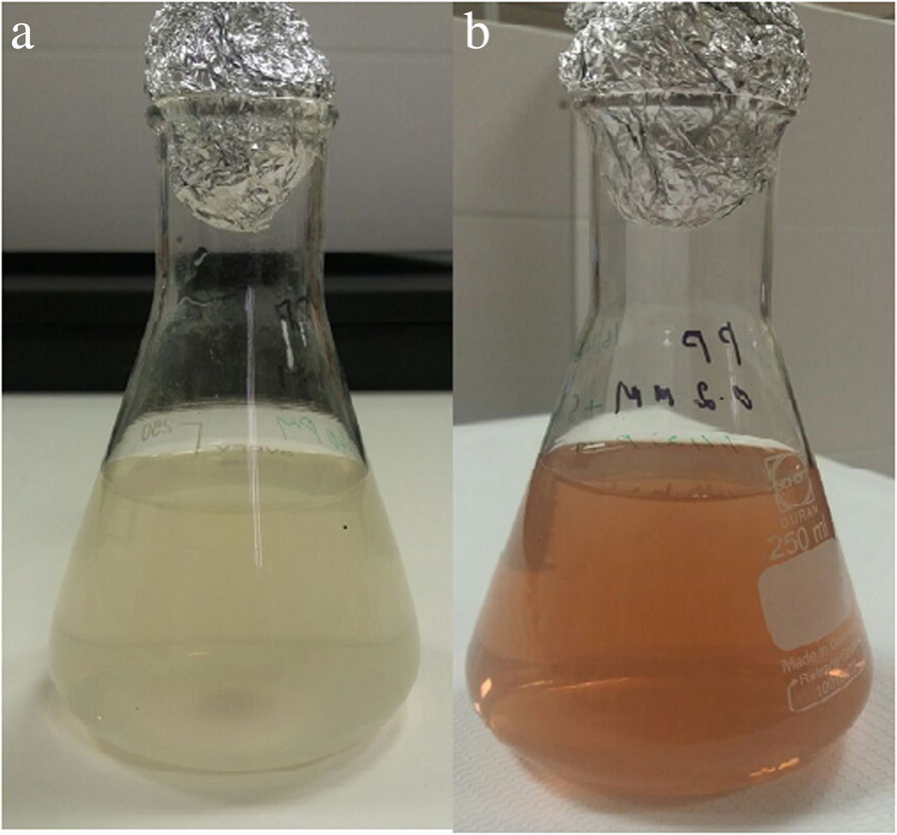
**a** 0.l mM of silver nitrate. **b** Color changes after *P. granatum* peel extract added

The AgNPs were successfully synthesized using the aqueous extract of pomegranate peel as a reducing agent source. Figure 1a shows 0.1-mM silver nitrate dissolved in 250 mL of DDH_2_O to make a colorless solution. Then, 10 mL of aqueous peel extract was added and mixed well, and the reaction mixture slowly changed to a dark brown color over 24 h, as seen in Fig. 1b. The color change observed during the synthesis of AgNPs has been reported for similar reactions when several types of plant part extracts such as leaves, flowers, peels, seeds, and fruits are used. The color change was due to AgNO_3_ interacting with plant sources and being reduced from silver nitrate to elemental silver [18–22].

**Fig. 2:**
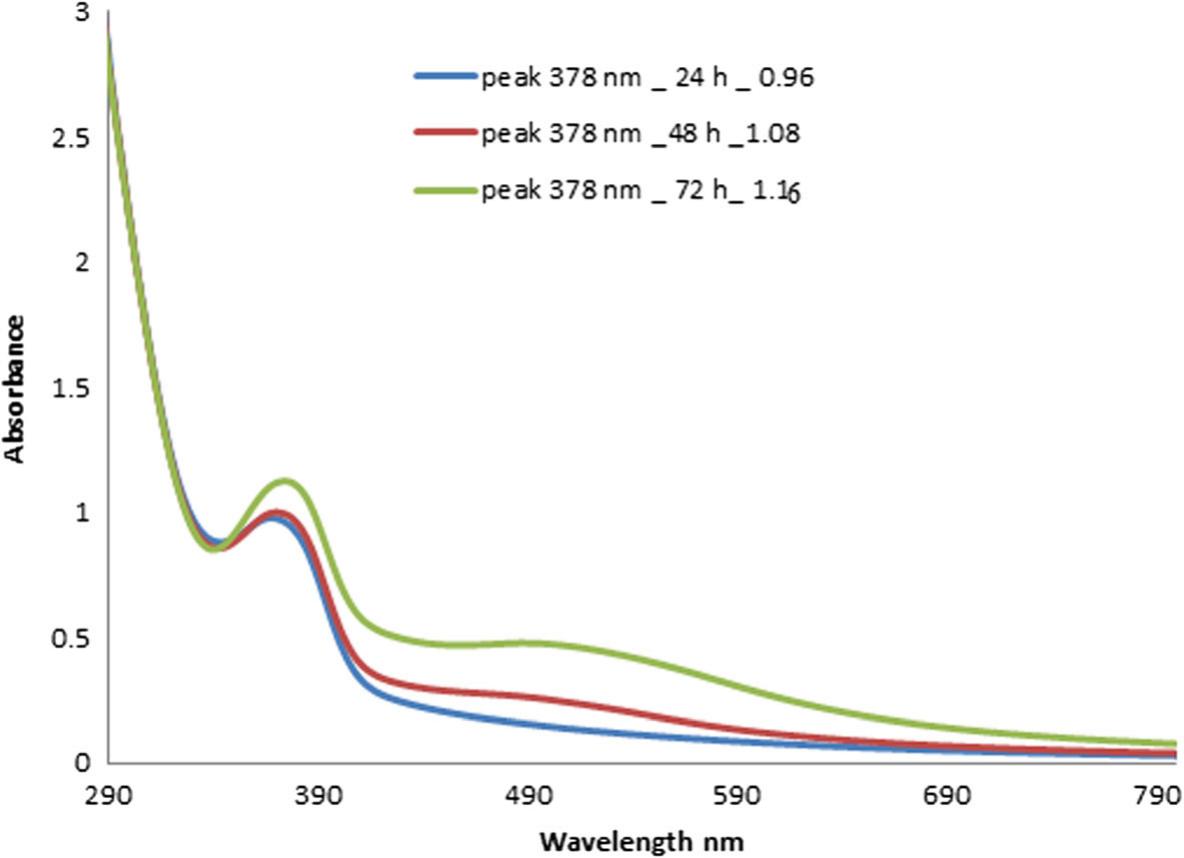
UV-Vis absorbance spectra of synthesized AgNPs at 48 to 72 h in time intervals

Figure 2 shows the UV-Vis spectrum of AgNPs synthesized using pomegranate peel aqueous extract. As shown in Fig. 2, the absorbance band has a peak at 378 nm at reaction times of 24, 48, and 72 h with intensities of 0.96, 1.08, and 1.16, respectively. The intensity increased with time, as the reaction had more time to occur, leading to higher concentrations of AgNPs. The surface plasmon resonance data showed that increasing concentrations of AgNPs led to increasing AgNP peaks, coinciding with increased amounts of reduced silver over time. As AgNO_3_ reacted to form AgNPs due to the release of electrons from the pomegranate extract, a concurrent reaction started to oxidize ascorbate radicals. A similar UV-Vis absorption spectrum was observed in a different study that produced AgNPs from pomegranate peel extract, with an absorbance peak at 371 nm [23].

**Fig. 3:**
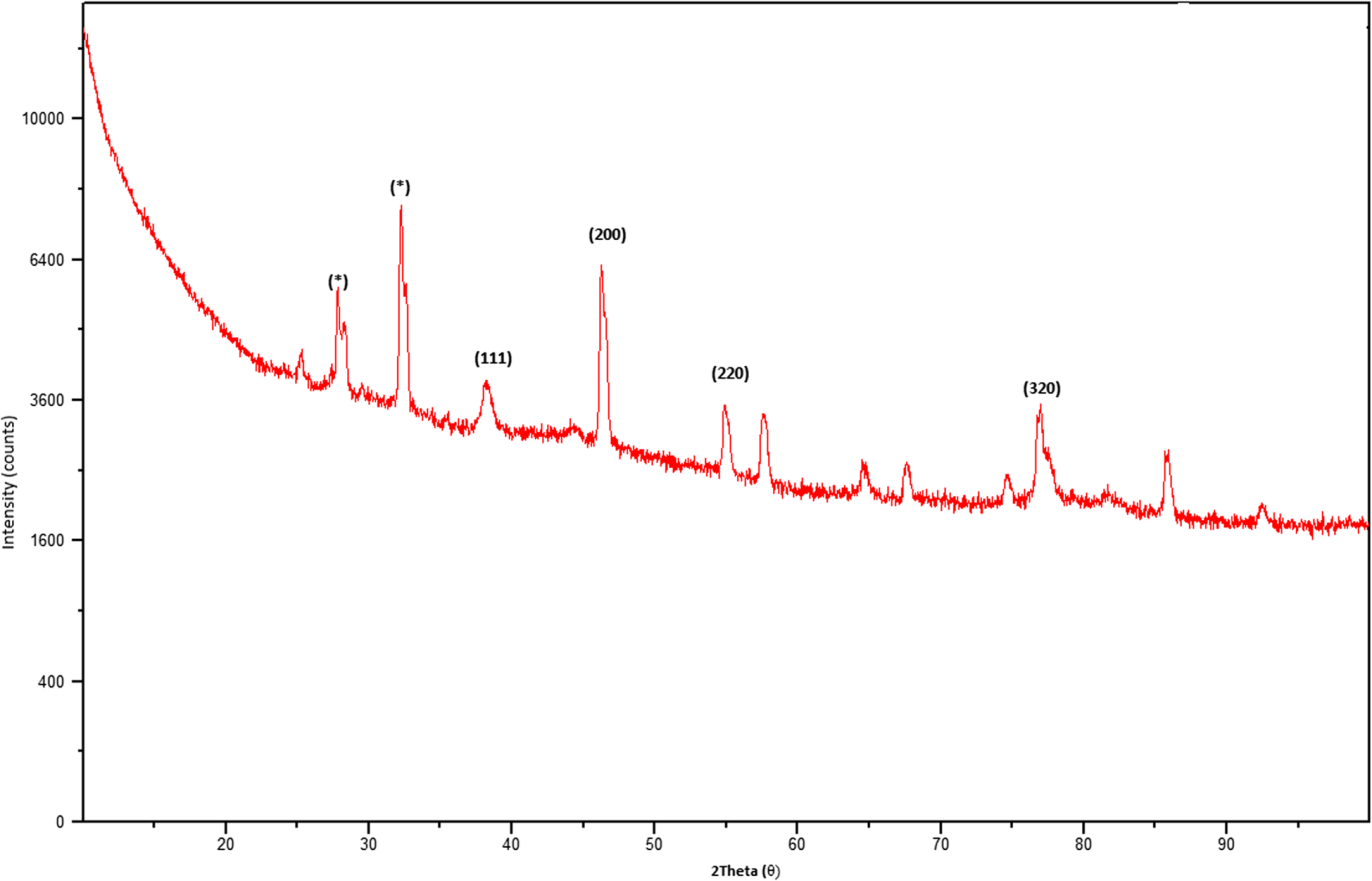
XRD pattern of synthesized AgNPs from *P. granatum* peel extract

**Fig. 4:**
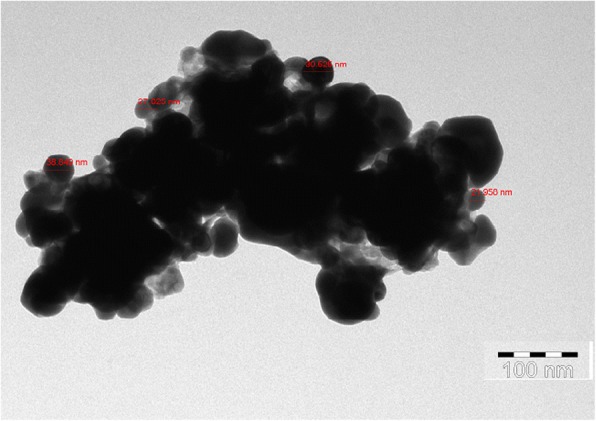
TEM image of synthesized AgNPs from *P. granatum* peel extract

**Fig. 5:**
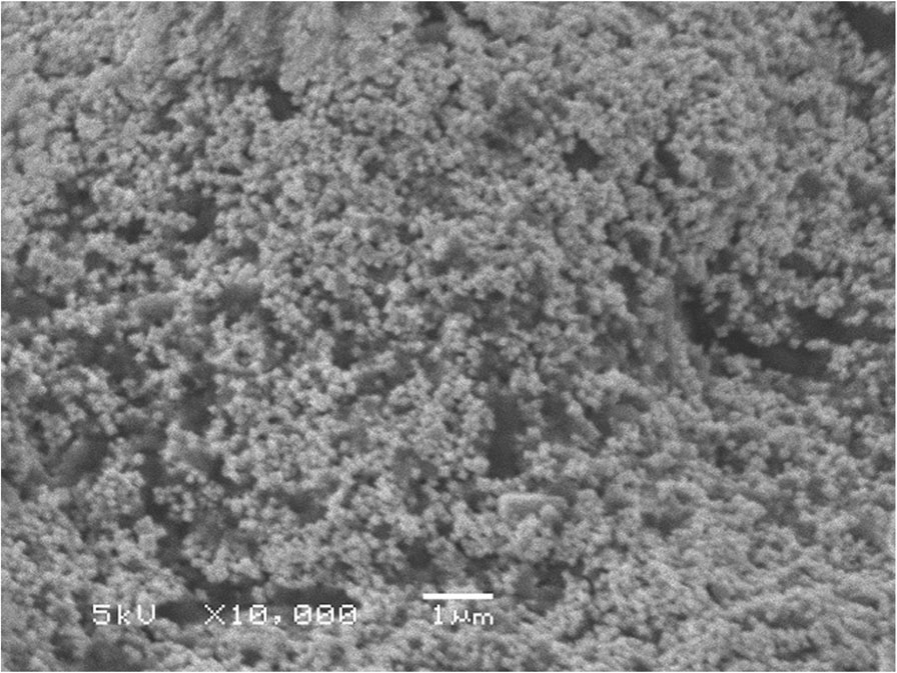
SEM image of synthesized AgNPs from *P. granatum* peel extract

The XRD pattern of green-synthesized AgNPs is shown in Fig. 3. Six intense diffraction peaks are observed at 2*θ* values ranging from 0 to 90, indicating that we could assign the 111, 200, 220, and 311 planes of a faced cube with a central Ag ion. The XRD spectrum suggests that the synthesized AgNPs formed into a crystalline structure. This result agrees with XRD patterns previously published in the JCPDS database (No. 04-0783). The unidentified crystalline peaks (*) observed correspond to silver oxides [24]. A TEM image of 0.1-mM pomegranate aqueous peel NPs is shown in Fig. 4. This image showed particles were spherical in shape with a diameter ranging from 20 to 40 nm, with the average particle size being 26.95 nm. Similar reports have been made regarding the nanosynthesis of NPs using *Actinidia deliciosa* fruit extract [25]. The SEM observations of the synthesized AgNPs (Fig. 5) show an equal distribution of silver nanoparticles on the surface of pomegranate peel cells. From this image, it was determined that the nanoparticles are spherical in shape, with diameters ranging from 20 to 40 nm, which is similar to a previous report of spherical shaped AgNPs ranging from 34 to 50 nm in diameter produced using *Raphanus sativus* L. peel extract [26].

In the phytosynthesis of silver nanoparticles using the pomegranate peel extract presented here, the size of the obtained nanoparticles is quite promising for drug delivery. The size of the nanoparticles being less than 100 nm is reported to play a role in the development of smart systems, enhancing the therapeutic and imaging values and drug delivery to specific tissues to provide controlled-release therapy [27]. The size and shape of the nanoparticles influences the bioavailability of drug in target tissues. Nanoparticles that are 100 nm are reported to exhibit 2.5-fold greater uptake when compared to that if 1-μm diameter particles [28, 29]. The size of the nanoparticles plays a key role in particle function, such as degradation, vascular dynamics, targeting, clearance, and uptake mechanisms [30]. Furthermore, the nanocrystalline nature of the synthesized AgNPs improves the bio-distribution and pharmacokinetics as reported [31, 32]. The utilization of pomegranate biowaste will be a novel approach towards waste utilization, as reported earlier [33].

**Fig. 6:**
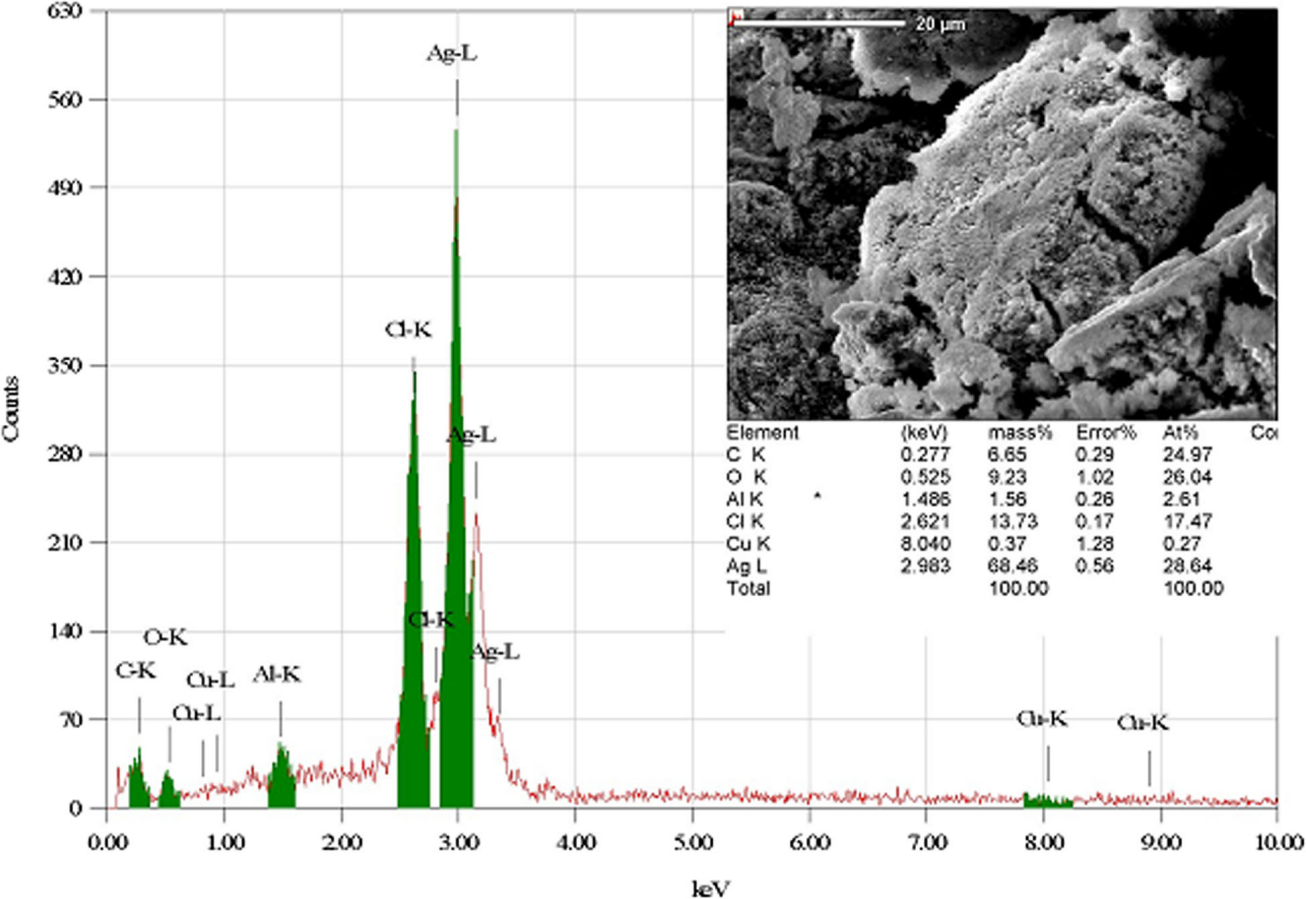
EDX image of synthesized AgNPs from *P. granatum* peel extract with quantitative analysis

Data from the EDX study provided a qualitative and quantitative analysis of the elements found in the synthesized nanoparticles, as shown in Fig. 6. The EDX study provided an elemental breakdown of the content of the synthesized NPs and estimated that the NPs consisted of 70% Ag by weight. Other elements and bonds identified in the results included C-K, O, C-U, Cu, and K, each of which corresponded to a small percentage of the total mass. The EDX report provides evidence that the low concentration of 0.1 mM AgNO_3_ resulted in high numbers of synthesized AgNPs. Similar results were reported for 0.3 mM AgNO_3_ that was poured into distilled water for 3 h and heated to 300 °C, and for 1, 2, and 3 g of pomegranate peel extract mixed with 30 mL of distilled water and heated to 80 °C [34].

**Fig. 7:**
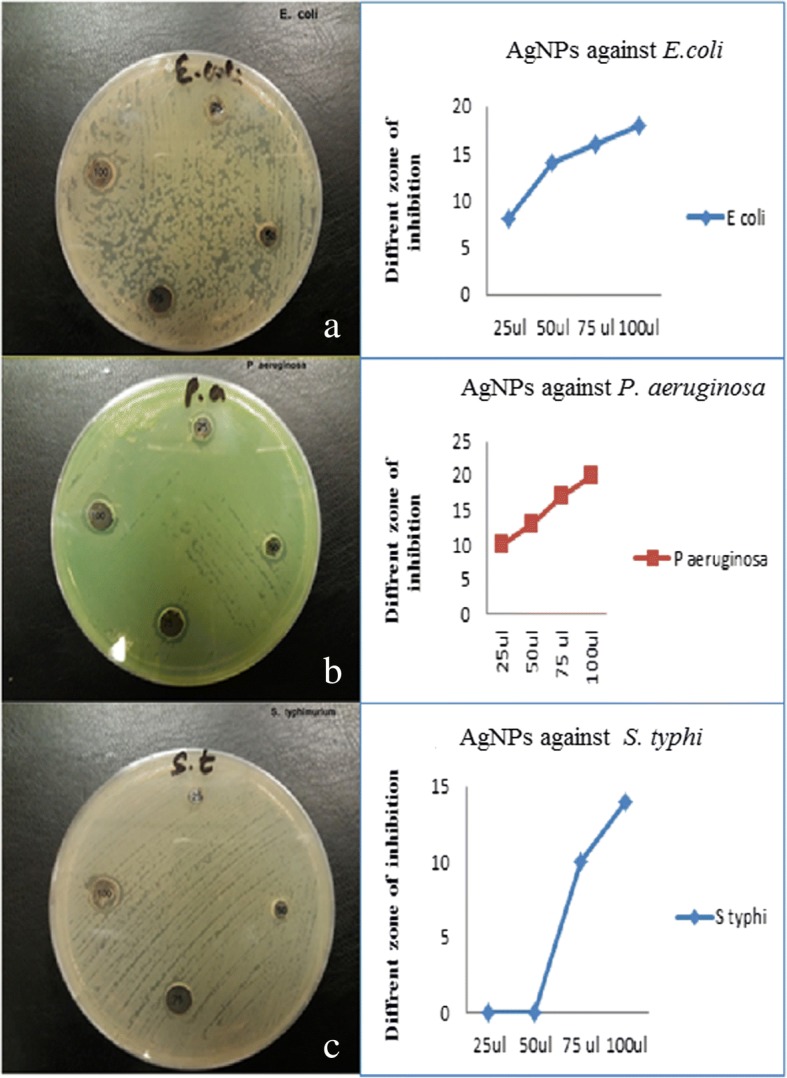
Antimicrobial effects and zone of inhibition of AgNPs of Gram-negative pathogens (**a**–**c**)

The antibacterial properties of the pomegranate synthesized AgNPs were investigated using 25, 50, 75, and 100 μg/mL samples against both Gram-positive and Gram-negative bacteria via the agar well diffusion test. Agar plates with the Gram-negative bacteria *E. coli*, *S. typhi*, *and P. aeruginosa* and the zones of inhibition are shown in Fig. 7a–c. Low concentrations of pomegranate synthesized AgNPs (25 and 50 μL) showed inhibitory activity against *P. aeruginosa* and *E. coli* but not against *S. typhi.* Similar antimicrobial effects of pomegranate products have been previously reported, where the strongest inhibitions were observed for *E. coli*, *S. aureus*, and *P. aeruginosa* [35–37].

**Fig. 8:**
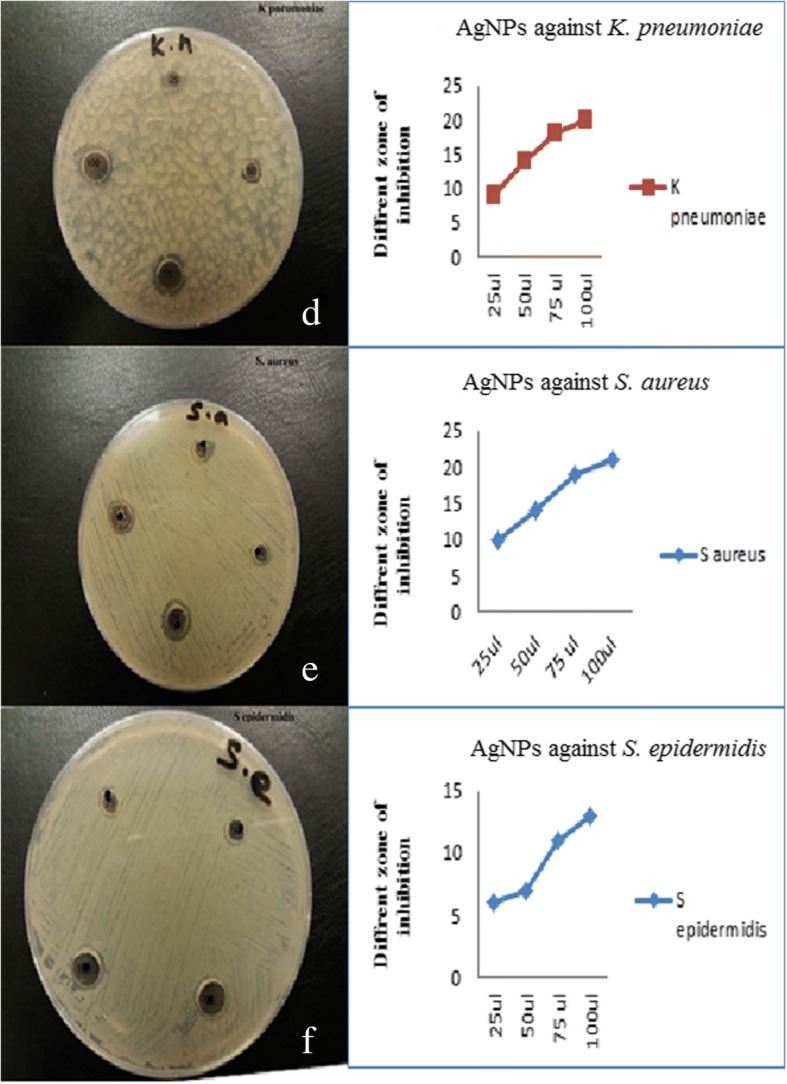
Antimicrobial effects and zone of inhibition of AgNPs of Gram-positive pathogens (**d**–**f**)

**Fig. 9:**
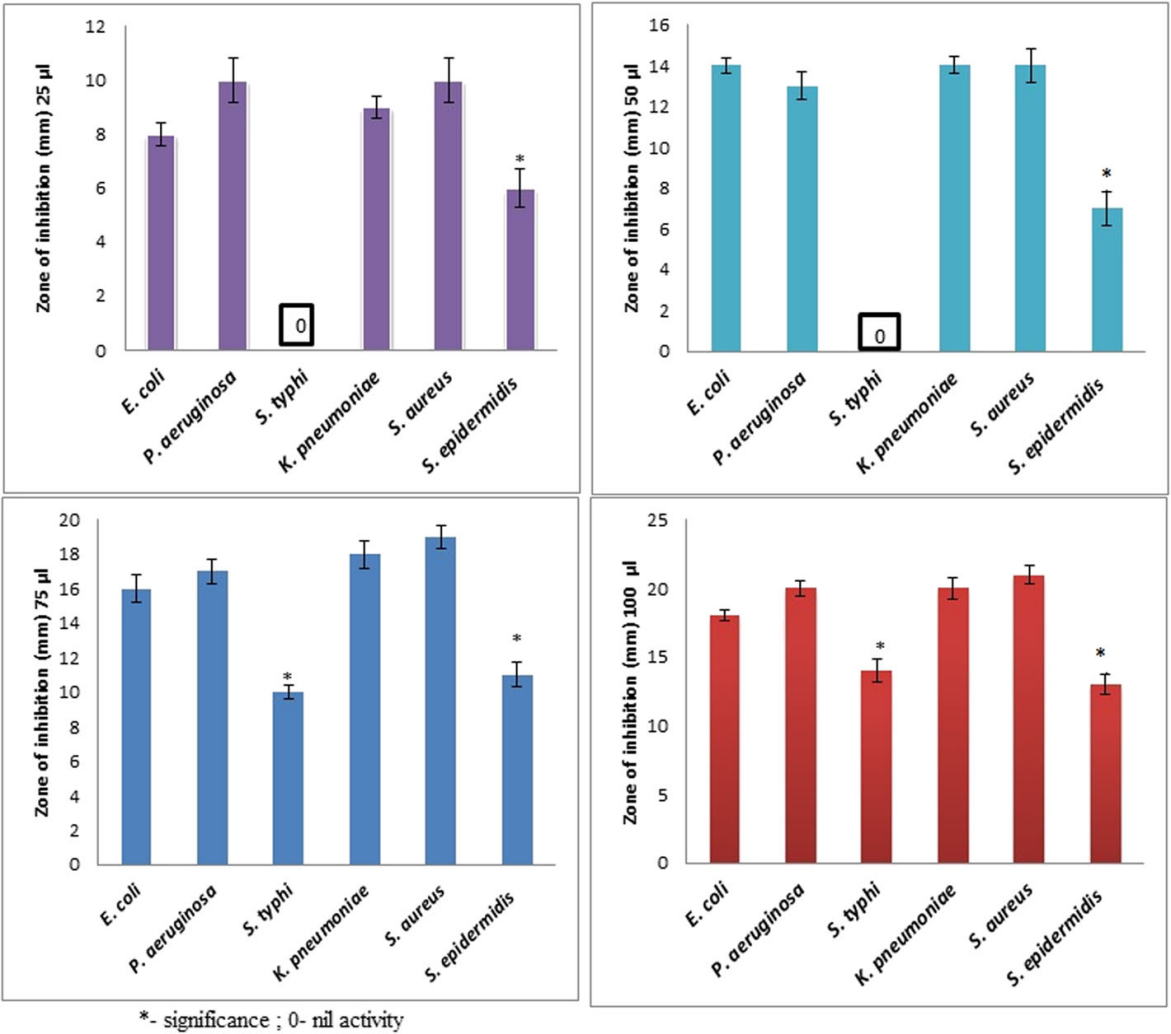
Antimicrobial activity of AgNPs against Gram-negative and Gram-positive pathogens

Figure 8a–c shows the antimicrobial activity of synthesized AgNPs against the Gram-positive pathogens *K. pneumoniae*, *S. aureus*, and *S. epidermidis*. Antimicrobial activity was observed even at low AgNP concentrations (25 and 50 μL) for *K. pneumoniae,* with zones of inhibition of 9 and 14 nm, respectively, and against *S. aureus*, with inhibition zones of 6 and 14 nm, respectively. Earlier studies also confirmed the growth inhibition of Gram-positive bacteria treated with synthesized NPs [35–38]. The antibacterial activity evaluated after exposure to synthesized AgNPs showed zones of inhibition in the range 7 to 21 mm. Figure 9 presents the inhibitory effects of different concentrations (25 to 100 μL) of *P. granatum* peel AgNPs on *E. coli*, *P. aeruginosa*, *S. typhi*, *K. pneumoniae*, *S. aureus*, and *S. epidermidis*. Even at low concentrations of AgNPs, a good antibacterial activity was observed for all microbes, except *S. typhi*, as reported earlier [38].

**Fig. 10:**
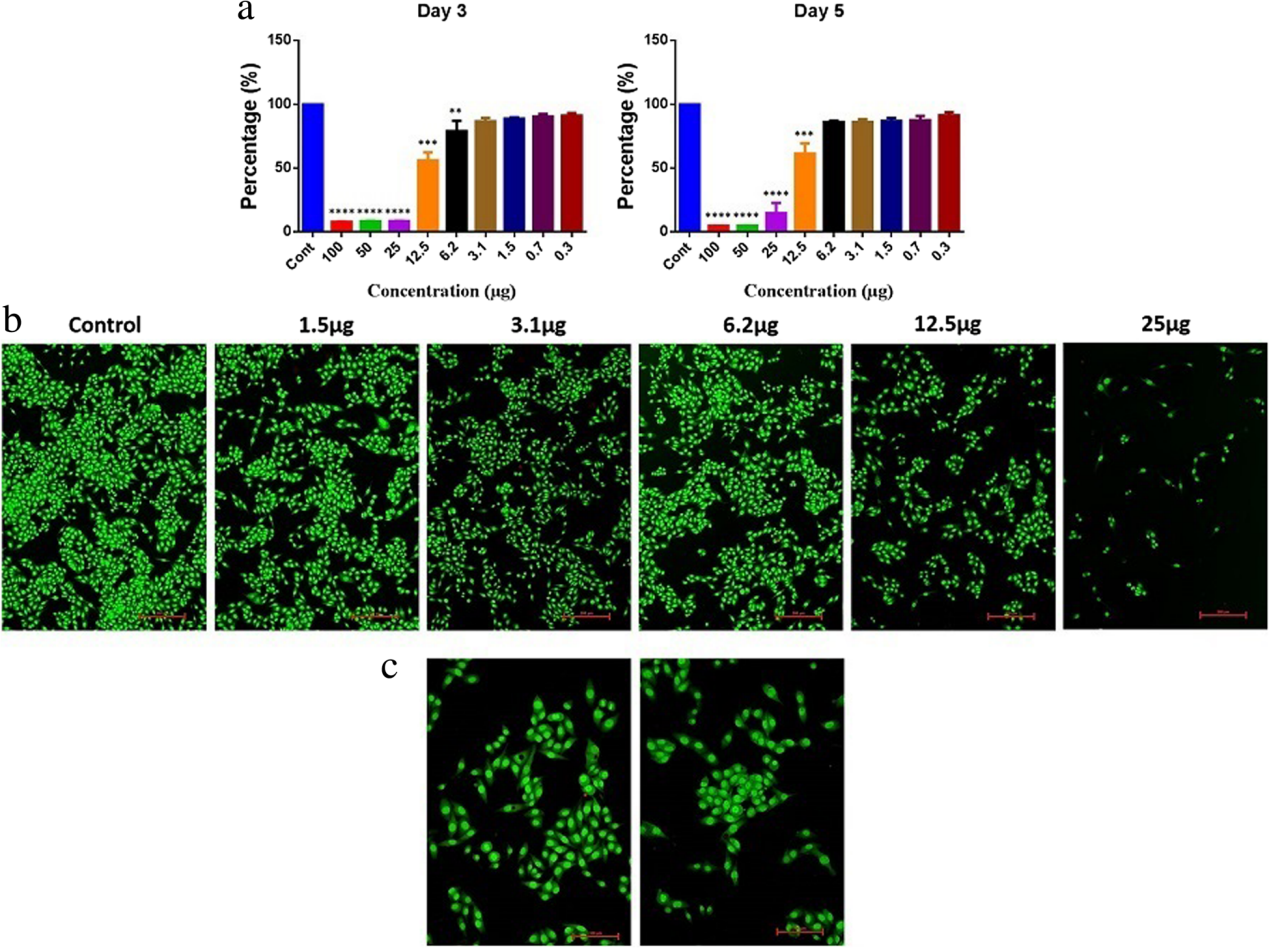
Cytotoxicity of AgNPs. **a** Cell proliferation and viability analysis on RKO cells. One-way ANOVA multiple comparisons, ***P < 0.0005. **b** Apoptosis/necrosis analysis on RKO cells. **c** RKO cells exposed to different doses of AgNPs

In order to analyze the cytotoxic effects of AgNPs, a colon cancer cell line (RKO: ATCC® CRL-2577™) was utilized. On day 3, we found a viability of 56% with a 12.5-μg treatment and a viability of 61% on day 5. The overall significant reductions in proliferation were observed at > 12.5 μg (Fig. 10a), and it was consistent on day 5. Furthermore, AO/EtBr stained images confirmed proliferation reduction by visualizing colonies and cell numbers (Fig. 10b). Interestingly, we could observe cells with perinuclear cytoplasmic vacuoles at 12.5 μg (Fig. 10b, c); this process might be a route of degradation in lysosomes in the process of autophagy for enhancing programmed cell death. However, further studies are needed to confirm the effect of AgNP on autophagy functions. In our previous study on AgNPs synthesized using *Pimpinella anisum* seeds, we also found that 12 μg of AgNPs was toxic to HCT116 cells by enhancing either apoptosis or necrosis [12]. A low concentration of AgNPs was also reported to be able to induce apoptosis [39]. The current experiments with AgNPs synthesized with *Punica granatum* peel extract also showed 55–62% toxicity with 12.5 μg. Moreover, AO/EtBr staining revealed a clear picture of programmed cell death via autophagy.

## Conclusions

The outcome of the present study showed that pomegranate peel extract is a good reducing agent to synthesize silver nanoparticles with a size range of 20–40 nm (average size, 26.95 nm), an ideal prerequisite for efficient drug delivery and for increased bioavailability at a target site. The antibacterial activity of the synthesized AgNPs on tested organisms, even at low concentrations of AgNPs (25–100 μL), further confirms the antibiotic efficiency of the green-synthesized AgNPs for the development of novel antibacterial agents for treatment against Gram-negative and Gram-positive pathogens. Moreover, the observed cytotoxic effects of AgNPs on colon cancer cell lines and the reductions in cell proliferation at a dose level of > 12.5 μg further promote AgNPs as a first-line treatment for tumors.

## Abbreviations

EDX: Energy-dispersive X-ray spectroscopy
SEM: Scanning electron microscope
TEM: Transmission electron microscopy
XRD: X-ray diffraction
